# The Importance of a Hierarchical Approach in Investigating the Connection Between Peripheral Artery Disease and Risk for Developing Low-Trauma Fractures: A Narrative Literature Review

**DOI:** 10.3390/jcm14051481

**Published:** 2025-02-23

**Authors:** Petar Milovanovic, Jelena Jadzic, Danijela Djonic, Marija Djuric

**Affiliations:** Center of Bone Biology, Faculty of Medicine, University of Belgrade, 11000 Belgrade, Serbia; drpmilovanovic@gmail.com (P.M.); jelena.jadzic@med.bg.ac.rs (J.J.); danijela.djonic@med.bg.ac.rs (D.D.)

**Keywords:** peripheral artery disease, PAD, bone fracture, osteoporosis, bone mineral density, hierarchical bone organization

## Abstract

Considering that skeletal changes are often asymptomatic during routine clinical examination, these disorders are frequently overlooked in patients with peripheral artery disease (PAD). Keeping in mind the inclining prevalence of PAD and bone fragility, especially in older individuals, this narrative literature review aimed to provide a comprehensive overview of skeletal alterations in patients with PAD, focusing on the importance of the multi-scale and multidisciplinary approach in the assessment of the bone hierarchical organization. Several observational studies have shown a connection between PAD and the risk of developing low-trauma fractures, but numerous ambiguities remain to be solved. Recent data indicate that evaluating additional bone properties at various levels of bone hierarchical structure may help in understanding the factors contributing to bone fragility in individuals with PAD. Further research on bone structural alterations (especially on micro- and nano-scale) may enhance the understanding of the complex etiopathogenesis of skeletal disorders in patients with PAD, which may lead to advancements in optimizing the clinical management of these individuals. Since osteoporosis and PAD have numerous overlapping risk factors, it is meaningful to evaluate vascular status in individuals with osteoporosis and examine bone health in individuals with PAD to identify individuals who require treatment for both diseases.

## 1. Introduction

Peripheral artery disease (PAD) is a primary cardiovascular disease. It is characterized by debilitating atherosclerotic occlusion of an artery or arteries, excluding the coronary and cerebral arteries [1,2]. Although PAD is defined quite broadly, the term is most often reserved for arterial disorders of the lower limb, including “asymptomatic lower limb PAD, intermittent claudication (IC), and chronic limb-threatening ischemia (CLTI)” [1,2]. Generally, patients with PAD may also have concomitant cardiovascular and/or cerebrovascular disorders, further increasing their mortality [2]. PAD could be asymptomatic for a while, but when symptoms develop, they cause a reduction in quality of life with severe limitations in physical function, particularly in walking and numerous daily activities [2]. According to the Global Peripheral Artery Disease Study, PAD is commonly defined using an ankle-brachial index (ABI) of 0.90 or less, representing the “ratio of the systolic blood pressure at the ankle to the systolic blood pressure in the arm” [2].

In patients with PAD, various skeletal alterations can occur, including infection [3], osteonecrosis [4], and metabolic bone disorders [5]. However, bone fractures, particularly low-trauma fractures, represent the most significant health burden [6]. Numerous observational studies have shown a connection between PAD and the risk of developing low-trauma fractures, but numerous ambiguities remain to be solved.

These two conditions have many overlapping risk factors, including nonmodifiable risk factors such as genetic predisposition and advanced age, and modifiable risk factors such as metabolic syndrome and its components (dyslipidemia, obesity, insulin resistance, hypertension), type 2 diabetes, and sedentary lifestyle [7]. While bone fragility and PAD were previously attributed to age-related changes, recent evidence has suggested that their association exceeds the mere process of aging [8]. One of the largest studies that investigated the correlation between PAD and bone fragility suggests that an association is present independent of the shared risk factors and that persons of both sexes with diagnosed osteoporosis may have an increased risk of developing atherosclerotic disease at an earlier age [9].

It is not yet sufficiently clear how arterial vascularization causes skeletal alterations. Therefore, it is essential to comprehensively investigate bone changes in individuals with PAD to clarify the connection between PAD and bone fragility so that appropriate and timely preventive and treatment strategies can be implemented to improve health outcomes in these patients. Thus far, there have been no comprehensive summaries of the available evidence on the relationship between PAD and bone fragility, especially in the context of various methods for the assessment of bone strength, including both routine methods (which provide overall, mostly macro-scale, assessment of bone mass and mineral content, and based on that, estimate the fracture risk) and more advanced evaluation techniques (which provide structural bone evaluation, deciphering the notable contribution of lower-scale levels of bone hierarchical organization on the overall bone strength). Thus, this narrative literature review aimed to provide a comprehensive overview of the contemporary literature on skeletal changes in patients with PAD, focusing on the importance of the multi-scale approach in assessing bone hierarchical organization.

## 2. Literature Search Strategy

An electronic search was conducted using the PubMed/Medline, Web of Science, Cochrane, and National Library of Medicine—National Institutes of Health databases in December 2024. Three authors independently obtained search results using the search terms given in Table 1 to identify published articles on skeletal alterations in patients with PAD. Considering that PAD diagnostic criteria varied over time, we applied an inclusive approach in this review, meaning that restrictions regarding the definition of PAD and its diagnostic criteria were not applied. Thus, we included all studies that characterized PAD as a debilitating atherosclerotic occlusion of an artery or arteries, excluding the coronary and cerebral arteries. The authors independently reviewed the obtained search results, and only human studies with at least an abstract written in English were included in this review. Differences in search results were addressed through discussions, and all authors reached a consensus regarding the final selection of articles included in this review.

## 3. Epidemiological Data

In recent decades, the prevalence of PAD, as one of the clinical manifestations of atherosclerosis, has been growing tremendously. The general prevalence of PAD has been reported to be between 3% and 10%, while in persons above the age of 70 years, the prevalence may even reach 20% [10]. Other studies suggest a wider prevalence range, from 6.3% to 21.4%, depending on age, sex, and PAD definition [11]. According to the Global Peripheral Artery Disease Study, at the global level, PAD affected more than 200 million individuals in 2010, and nearly 70% of them were residents of low- and middle-income countries [12]. More recently, the Global Peripheral Artery Disease Study estimated that, in 2015, around 236 million people had PAD worldwide, with a slightly higher percentage of women affected [2].

There is still limited epidemiological data about various fracture types in patients with PAD, and available evidence is limited and inconsistent. While some studies have not reported significant associations between PAD and fracture occurrence [13], other studies have shown an increased risk of fractures in individuals with PAD. For example, two large cohort studies in Taiwan have shown that the incidence of fractures in people with PAD was 22.1 per 1000 person-years, compared with 15.5 per 1000 person-years in people without PAD [14]. Further stratification of the fracture risk by the skeletal site has shown that PAD was associated with an increased risk of fractures of the upper limb (hazard ratio [HR] = 1.56), neck or trunk (HR = 1.52), and lower limb in general (HR = 1.44) and hip in particular (HR = 1.48) [14]. Another study has shown that the risk of major osteoporotic fracture determined by FRAX was negatively associated with vascular function (assessed by flow-mediated vasodilation and nitroglycerine-induced vasodilation) and positively associated with brachial artery intima-media thickness in both men and women [15], suggesting the importance of evaluating bone health in individuals with PAD, as well as evaluating vascular status in patients with osteoporosis. As one of the most common and most detrimental low-trauma fractures, hip fractures have attracted greater attention from researchers in the context of PAD.

Namely, a systematic review with meta-analysis was conducted on the articles published until October 2017 in the MEDLINE and EMBASE databases, specifically the cohort and case-control studies that investigated the effects of PAD on the risk of subsequent hip fracture [16]. A total of six eligible cohort studies were identified, which comprised almost 16,000 patients with PAD. The results of the meta-analysis showed a significant association between PAD and incident hip fracture (relative risk [RR] = 1.64), both in prospective (RR = 1.60) and retrospective studies (RR = 1.72). However, the study emphasized high inter-study heterogeneity and possible publication bias as major limitations [16].

In the Health in Men Study [17], which included more than 4000 individuals in whom ABI was measured, claudication was not an independent predictor of subsequent hip fracture, but a significant association was found between PAD (ABI < 0.9) and fracture occurrence ([HR] = 1.69).

The Swedish MrOS (Osteoporotic Fractures in Men) study [18], a prospective study of Swedish elderly men, followed more than 3000 men over 10 years. During that period, less than 200 individuals experienced an incident hip fracture [18]. PAD was associated with an increased hip fracture risk (HR = 1.70), after adjusting for age and study site. Even after adjusting for total hip BMD, the significant association remained (HR = 1.64). Full adjustment (i.e., age, site, hip bone mineral density [BMD], body mass index [BMI], falls, smoking, estimated glomerular filtration rate [eGFR], former hip fracture, handgrip strength, walking speed, antihypertensive treatment, diabetes, history of cardiovascular disease, and education) further reduced the HR (HR = 1.38) [18].

## 4. Osteodensitometric Evidence for the Relationship Between PAD and Bone Fragility

Previous studies have provided evidence for the potential association between PAD and bone health. These studies differed in the examined population (different geography, race, age, sex, and presence of other diseases), sample size (from less than 20 to more than 100,000 individuals), study design (retrospective, cross-sectional, prospective), measured indicators of bone health (BMD and T score at different skeletal sites, but most often at the hip region), and methods used to assess vascular status and/or define PAD.

In the following text, we provide brief specific summaries of the evidence from a number of previous studies on this subject. Initial small-scale cross-sectional studies in France (with less than 20 patients, only men) have shown lower BMD at the femoral neck of individuals with PAD [19], and significantly lower bone mineral content (BMC) on the side of the leg with more severe PAD as assessed by Doppler ultrasonography [20]. Most further studies assessed vascular status based on ABI, where some examined the correlation between ABI and bone parameters without clearly defining PAD on the basis of a certain ABI threshold, while other studies clearly defined PAD as ABI <0.9 or ≤0.9:

**Correlation between ABI and osteodensitometric parameters without defining the threshold for PAD:** Vogt et al. examined the associations between ABI and BMD measured at the axial and appendicular skeleton of 1292 community-dwelling elderly women from the Study of Osteoporotic Fractures in the US [21]. After adjustment for age, the authors found weak but positive associations between ABI and BMD at the radius, calcaneus, and hip, but not the lumbar spine. History of diabetes did not affect these associations, but adjusting for smoking and BMI did explain approximately 50% of the association at the hip. In the prospective part of the study, the authors showed a greater decrease in BMD at the hip and calcaneus in women with a greater annual decrease in ABI, which was independent of other factors such as smoking, BMI, diabetes, and exercise [21]. A large prospective cohort study was performed by Wong et al. in Hong Kong, including almost 4000 older men and women [22]. In that study, ABI positively correlated with hip BMD, but the association was heavily affected by confounding factors such as age, sex, body weight, smoking status, and diabetes [22].

**Relationship between PAD (defined as ABI <0.9 or ≤0.9) and osteodensitometric parameters:** In the Rotterdam Study, Van der Klift et al. performed a cross-sectional evaluation of the association between PAD (defined as ABI < 0.90) and BMD in more than 5000 older adults (3053 women and 2215 men) [23]. After adjusting for age, PAD was associated with lower BMD at the femoral neck in women, but not in men. Neither sex showed a significant association between PAD and BMD at the lumbar spine [23]. In Italy, Mangiafico et al. conducted a cross-sectional study in postmenopausal women, including 345 ambulatory osteoporotic patients and 360 community-based age-matched patients with normal BMD [24]. PAD (defined as ABI < 0.90) was significantly more common in women with osteoporosis than in women with normal BMD, and mean ABI was significantly lower in the osteoporosis group. T score at the femoral neck was significantly lower in osteoporotic patients with PAD than in those without, independent of other recorded factors such as BMI and smoking [24]. In the Rancho Bernardo Study in the US, the authors examined the association between PAD (defined as ABI ≤ 0.90) and bone health in a cohort of more than 1300 community-dwelling older adults in Southern California [13]. The study showed a significantly lower BMD at the femoral neck in men with PAD and a significantly greater decrease in BMD at the total hip in women with PAD compared with men/women without PAD. The prevalence of osteoporosis at the femoral neck and total hip was significantly higher in the presence of PAD in women but not in men. However, adjustment for age abolished the significant skeletal effects of PAD in this study, whereas adjustments for BMI, habits (exercise, smoking), comorbidities (hypertension, diabetes), and biochemical parameters (cholesterol/HDL ratio, creatinine clearance) did not affect the results [13]. More recently, Zhang et al. performed a cross-sectional study on 272 elderly men with confirmed type 2 diabetes, including 222 patients without PAD and 50 patients with subclinical PAD (defined as ABI ≤ 0.9), in China [25]. They found a significantly lower total hip BMD and T score in the group with subclinical PAD, and confirmed subclinical PAD as an independent risk factor for the reduced BMD at the total hip [25]. In a prospective cohort study in six centers in the US [26], including almost 6000 community-dwelling elderly men, the annual decrease in BMD at the total hip and subregions of the hip, such as femoral neck and trochanter, was greater in the PAD group (defined as ABI <0.9) than in the non-PAD group after adjustment for age, race, site, and baseline BMD. Further adjustment for multiple confounders partially weakened the observed difference. There was also a higher incidence of non-vertebral fracture in the PAD group [26]. Based on the data obtained from Life Line Screening Inc., Baldwin et al. showed a strong association between lower BMD and PAD (defined as ABI ≤ 0.9), even after adjusting for age and atherosclerotic risk factors. Specifically, in men, osteopenia or osteoporosis was associated with a significantly higher prevalence of PAD (4.5% or 10.9%, respectively) compared with men with normal BMD (3.0%) [9]. In women, osteopenia or osteoporosis was associated with a significantly higher prevalence of PAD (4.8% or 11.8%, respectively) compared with women with normal BMD (3.3%) [9]. In the Swedish MrOS study [18], a cohort study, PAD was defined based on ABI (<0.9). After adjusting for total hip BMD, there was still a significant association between PAD and hip fracture risk, suggesting that the increased hip fracture risk is partially independent of BMD [18].

While most studies examined vascular status based on functional indicators, typically by ABI, morphological evaluations were also considered in some studies. Indeed, some studies assessed vascular status based on ultrasonic verification of atherosclerosis and/or calcifications, radiographic evaluation of vessel calcifications, and even angiography.

Association between osteodensitometric parameters and vascular status assessed by ultrasonic verification of atherosclerotic lesions and/or vascular calcifications: Intending to clarify the relationship between vascular and skeletal alterations, Pennisi et al. conducted a cross-sectional study and examined 20 male and 16 female patients with PAD (defined by the ultrasonic verification of atherosclerotic lesions and/or vascular calcifications) and 30 age- and gender-matched healthy individuals in Italy [27]. The study revealed significantly lower age-adjusted BMD at the proximal femur and lumbar spine in the PAD group, independent of gender and diabetes. Based on parallel evaluation of bone turnover markers, the authors suggested reduced bone formation rather than increased bone resorption as the mechanism for the BMD decline [27].

Association between osteodensitometric parameters and vascular status assessed by radiographic assessment of vascular calcification: In a study on 963 older women in Denmark, Tanko et al. showed weak negative correlation between the radiological severity of aortic calcification and age-adjusted BMD at the proximal femur, but not the radius or spine [28]. A recent nationwide study in the US with almost 3000 patients has reported that greater severity of calcification in the abdominal aorta (evaluated from lateral DXA images of the lumbar spine) was associated with low femoral neck BMD, further suggesting the need to evaluate bone health in individuals with aortic calcification [29].

**Association between osteodensitometric parameters and angiographically confirmed PAD:** In a study examining 95 men and women with angiographically confirmed PAD and 44 control cases in Austria, Fahrleitner-Pammer et al. found a significant relationship between PAD and lower BMD at the femoral neck, independent of BMI and other factors [30]. Patients with PAD, especially those with local ischemic ulcers, showed signs of increased bone resorption [30].

In light of the hypothesis that a more comprehensive evaluation of vascular status is needed to assess the relationship between PAD and bone health more reliably, some studies assessed vascular status based on multiple methods, often showing that some of these methods correlated better than other with bone health indicators.

**Association between osteodensitometric parameters and vascular status assessed by multiple methods:** In a cross-sectional study in a healthy Chinese population (1467 men and 1020 women), the relationship between BMD and subclinical atherosclerosis was examined [31]. Brachial-ankle PWV showed a weak but significant association with BMD at the lumbar spine, particularly in females and especially after menopause, even after adjusting for confounding factors. However, ABI was not associated with BMD of the lumbar spine [31]. Another cross-sectional study conducted in 333 older men and 421 postmenopausal women in rural China did not show significant differences in BMD at the lumbar spine, femoral neck, and total hip between the cases with subclinical atherosclerosis (defined as cIMT ≥ 0.9 mm, the presence of carotid plaques, brachial-ankle PWV ≥ 1400 cm/s, and ABI ≤ 1) and the control group [32]. However, after adjusting for various confounding factors, only baPWV showed a borderline association with low BMD at the femoral neck in postmenopausal women [32]. Another study followed up more than 3000 individuals from the Cardiovascular Health Study for more than 10 years and examined the correlations between fracture incidence and several vascular indicators (baseline cIMT, aortic wall thickness, ABI) [33]. The study showed that despite a positive association with BMD, increased cIMT was associated with an increased hip fracture risk (HR = 1.18). However, aortic wall thickness and ABI did not correlate with hip fracture risk or BMD (available for a subset of patients) [33].

Finally, some studies did not define PAD directly, but rather relied on the ICD codes of PAD as recorded in the nationwide insurance databases.

**Association between osteoporosis and PAD as recorded in the insurance database:** In a longitudinal follow-up population-based study based on Taiwan’s National Health Insurance research database [34], which included more than 54,000 patients divided into the osteoporosis group and non-osteoporosis group, the risk of developing PAD was almost 30% higher in the osteoporosis group than in the non-osteoporosis group in both men and women [34]. In a more recent study on the same database, which included almost 130,000 patients divided to the osteoporosis group and non-osteoporosis group, the osteoporosis group showed a significantly higher risk of developing PAD over the 10-year follow-up period [35]. In these studies, PAD was defined as “at least three different medical statements issued in an out-patient setting or at least one claim in an in-patient setting” recorded in the database under the ICD codes for PAD [35].

**Summary of the osteodensitometric evidence for the relationship between PAD and bone fragility:**

In summary, critical analysis of osteodensitometric evidence for bone fragility in individuals with PAD reveals the following major points:

(1) There is a relationship (though inconsistent) between PAD and bone fragility: Namely, although most studies imply a relationship between PAD and bone fragility, or between vascular parameters and bone fragility indicators, there is considerable heterogeneity in the literature regarding this relationship, and some studies failed to identify any significant relationship.
(2) There seems to be a sex-specific pattern in the relationship between PAD and bone fragility; specifically, if a study included both men and women, the associations between PAD and bone fragility were usually stronger in women.
(3) The relationship between these two diseases is, to some extent, also influenced by other confounding factors, such as age, BMI, physical activity, smoking, and diabetes; indeed, despite the reported associations between PAD and BMD, multivariate analyses have often shown that the effects of PAD are not independent of other patient characteristics such as age and/or BMI, and in some studies, full adjustment for various relevant indicators completely abolished the previously significant association. This means that bone deterioration may be a consequence of aging or proinflammatory body composition rather than PAD itself, but also that aging and BMI drive both PAD and reduction in BMD. Likewise, diabetes, smoking, and low physical activity, all important cardiovascular risk factors, could promote PAD; however, diabetes probably has some vasculature-independent effects on bone deterioration, and smoking, low physical activity, and sarcopenia are generally associated with increased bone fragility. Hence, given the shared risk factors between PAD and bone fragility, it is often hard to segregate the specific effects of PAD on bone health. Nevertheless, in some studies, a significant impact of PAD on BMD remained even after thorough adjustment for confounding factors, suggesting that evaluation of bone health is reasonable in patients with PAD, even more so if we consider that most patients with PAD have other risk factors that may influence bone health (such as diabetes, smoking, and obesity), but are still relatively neglected in routine clinical evaluation.

Considering these major points, the source of heterogeneity of evidence may lie in various factors, such as differences in population characteristics, different skeletal sites examined (hip, spine, other sites), different statistical approaches with adjusting or not adjusting for various relevant confounding factors, and different definitions of PAD. As for the definition of PAD, most of the studies based the diagnosis of PAD on ABI (ABI < 0.9), but some studies have also measured other indicators such as cIMT, aortic wall thickness, calcification degree, and PWV, or even performed angiographic verification of PAD. While ABI is a clinically relevant indicator, it does not reflect whether vascular narrowing is particularly pronounced in some areas of the vascular tree, meaning that its use as a sole indicator of PAD may not provide sufficient information for linking the lesion in a specific artery with the skeletal sites supplied by that artery. This may partly explain the relatively low or no association between PAD and BMD (especially of the spine) in some studies. Other vascular indicators, such as cIMT, aortic wall thickness, calcification degree, and PWV, provide additional, different information about the specific parts of the vascular system or specific functional characteristics of the vascular tree, which may explain why some studies showed differential associations between bone indicators and various types of vascular indicators. Therefore, to provide more reliable conclusions, future studies should consider various vascular indicators so as to identify the optimal indicators that could be used in clinical assessment in the context of the association between PAD and bone health.

As evident from the illustrated studies, clinical assessment of the fracture risk was relatively rarely based on the incident fractures and mostly relied on DXA-based indicators such as BMD and T score. However, considering the results from the MrOS Swedish study, which also showed BMD-independent effects of PAD on hip fracture risk, and given the growing evidence that BMD and T score have limited ability to fully reflect fracture risk, especially in individuals with various diseases, it is important to examine other aspects of bone strength that are not captured by DXA technology.

## 5. The Importance of Multi-Scale Bone Assessment in Patients with PAD: A Promising Research Direction for the Future

**Figure 1:**
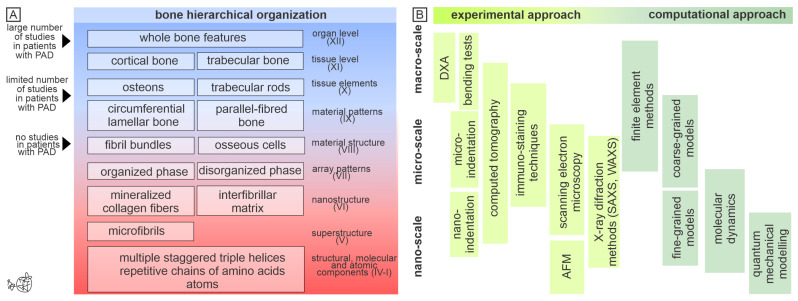
A variety of methodological approaches are available for multi-scale assessment of bone hierarchical organization in patients with peripheral artery disease. The left part of the figure illustrates the importance of the hierarchical bone organization (**A**), highlighting the difference between previously assessed bone characteristics and those that require further investigation in patients with PAD. The right part of the figure illustrates up-to-date methodologies that could be used to assess bone fragility in patients with PAD (**B**), highlighting the importance of experimental and computational approaches in the multidisciplinary assessment of bone hierarchical organization. The figure represents the author’s original work generated using vector graphic editor software (Corel DRAW, ver. 2021, for Windows operative system), signed by the creator. Abbreviations: PAD—peripheral artery disease; DXA—dual-energy X-Ray absorptiometry; AFM—atomic force microscopy; SAXS—small-angle X-Ray scattering; WAXS—wide-angle X-Ray scattering.

Besides external mechanical load characteristics, bone fracture occurrence depends on the internal bone features (internal factors) and their mutual interaction. It has been known that a considerable number of patients who experienced bone fractures have normal BMD [36], suggesting that DXA-generated BMD and T score cannot fully explain individual fracture susceptibility, meaning that other bone characteristics (on different levels of a bone hierarchical organization) contribute to increased fragility [37,38,39]. According to Reznikov et al., bone is organized in 12 levels of bone hierarchy [40]. As shown in Figure 1, the entire bone (level XII) consists of both trabecular and cortical bone (level XI), with human compact bone undergoing osteonal remodeling and featuring osteons (level X). The matrix of osteonal bone is made up of lamellae (level IX), which include mineralized collagen fibril bundles (level VIII) as well as organized and disorganized collagen fiber arrangements (level VII). The mineralized collagen fibril (level VI) comprises collagen and mineral particles. Collagen fibrils consist of microfibrils (level V) arranged in a quasi-hexagonal packing, with each microfibril formed from multiple staggered triple helices (level IV), which are in turn constructed from repetitive chains (level III) of amino acids (level II) containing atoms (level I). It is important to note that each of the hierarchical bone characteristics could be assessed by various up-to-date experimental and computational techniques, as shown in Figure 1.

For example, X-Ray imagining modalities (micro-CT or synchrotron radiation CT) could assess cortical and trabecular micro-scale bone features (bone trabecular micro-architecture, cortical porosity, bone vascular channels, and osteocyte lacunar network) [37,41,42]. Backscattered electron imaging is a very informative method for assessing bone mineralization, bone nanoporosity, and morphology of osteocyte lacunar network [43]. The functionality of bone cells (the expression of various cell-specific antigens involved in bone turnover regulation and intercellular communication) could be assessed by immunostaining methodologies (immunohistochemistry or immunofluorescence) [44], while alterations in bone collagen could be assessed by atomic force microscopy [37,45]. Given that bone mass correlated with the mechanical bone properties [39], computation methodologies are very valuable methods for bone strength simulation studies (Figure 1) [46]. However, it is indicative that a multidisciplinary approach (combining the results obtained from different experimental and simulation studies, Figure 1) is imperative to get a profound understanding of skeletal alterations in aging and diseases [47,48]. Thus, a multidisciplinary approach in hierarchical assessment of bone structure is a promising direction for future studies of bone fragility associated with PAD.

Considering that BMD and T score are limited by the two-dimensional nature and low resolution of DXA, inability to evaluate the internal bone organization, and inability to omit the interference of surrounding tissues (adipose tissue or vascular calcifications) at the imaging site, several attempts have been made to improve clinical fracture risk assessment [49]. For example, high-resolution peripheral quantitative CT (HR-pQCT) is a noninvasive 3D method for clinically assessing bone micro-architecture at the distal radius and tibia [50,51]. Further, HR-pQCT could be used to simultaneously evaluate arterial calcifications and micro-scale bone features, making a solid ground for a better understanding of osteo-vascular interactions in patients with PAD [52]. As shown in Table 2, contemporary literature suggests that micro-architectural alterations could contribute to reduced bone strength in individuals with PAD. Most of these studies have shown some association between PAD and micro-architectural decline (Table 2), but the variability in the data is worth highlighting. This variability primarily stemmed from a relatively small number of patients included in the studies, the single ethnicity of the study sample, various methods used to define PAD, and including individuals with different chronic comorbidities without systematic control for confounding variables (Table 2). Applicability of HR-pQCT is challenged by its high costs, unavailability, and the inability to assess clinically relevant fracture sites (e.g., lumbar vertebrae and femoral neck) [53], indicating the need to use other state-of-the-art methodologies to investigate skeletal alterations in patients with PAD. Importantly, future studies (using a multidisciplinary approach) should focus on distinguishing the independent contribution of trabecular and cortical compartments [54] in bone fragility of individuals with PAD.

Since contemporary literature lacks data about small-length (micro- and nano-scale) bone alterations in individuals with PAD (Figure 1), future studies should go beyond the state-of-the-art by analyzing the local microcirculation network at relevant fracture sites (e.g., femoral neck and lumbar spine), to provide direct local cues to the deteriorated microcirculation and allow for the more direct assessment of the correlation between bone structural, compositional, and mechanical characteristics and the alterations in bone microcirculation. Further, we believe that future studies should concentrate on the 3D structural and functional assessment of bone architecture, functional assessment of bone cells, structural assessment of mineral (hydroxyapatite crystals) and organic bone matrix components (bon collagenous and non-collagenous proteins), and functional assessment of bone marrow adiposity and bone vasculature to elucidate their roles in skeletal alterations of patients with PAD. The value of these studies could be enhanced by using multiple advanced methods to assess various characteristics of the same bone specimen from one patient [37]. Integrating clinical data with a multi-scale approach to hierarchical bone assessment can lead to developing patient-specific diagnostic algorithms to predict fracture risk associated with PAD. Examining bone hierarchical characteristics depends on evaluating the effects of various comorbidities in patients with PAD (e.g., type 2 diabetes, metabolic-associated liver disease, and obesity) to contribute to the mechanistic evaluation of bone fragility in these individuals.

## 6. Biological Basis for the Relationship Between PAD and Bone Fragility

Despite the relative breadth of clinical studies investigating the association between PAD and bone fragility, a detailed understanding of the biological mechanisms underlying this relationship is still lacking, given the insufficient evidence from microscopic studies of bone in individuals with PAD (Figure 1). Nevertheless, there is some experimental evidence for the effects of ischemia on bone metabolism. Namely, local hypoxia inhibits osteoblast differentiation and activity, thereby reducing bone formation, and increases osteoclastogenesis and osteoclastic activity, thereby favoring bone resorption [61,62,63]. These effects of hypoxia involve the local bone changes in energy metabolism, acid–base balance, and oxidative stress [64]. Moreover, hypoxia favors osteocyte cell death [65], followed by mineral impregnation of osteocyte lacunae, eventually leading to reduced mechanical competence of bone and increased bone fragility [66]. However, hitherto no clear consensus has been reached regarding the response of bone to hypoxia in the context of physiological and pathophysiological conditions in humans. According to a systematic review of hypoxic conditioning, there is no unified bone response to hypoxia; namely, sustained and intermittent hypoxia promotes osteoclast function and inhibits osteogenic differentiation, whereas cyclical hypoxia may exhibit the opposite effects [67], thereby even eliciting an anabolic effect [68]. Indeed, the effects of hypoxia on bone remodeling are very complex and are mediated through hypoxia-inducible factors (HIFs), and the contradictory findings may be explained by the different roles of HIF-1 and HIF-2, their different relative proportion in the local environment, and differences in age and bone cycle [64]. Still, more basic research is needed to better understand the effects of ischemia and hypoxia on bone in various contexts.

## 7. Limitations

This review has several limitations. First, it was restricted to studies with at least an abstract published in English. While this may have introduced some publication bias, we think that it was minimal given that even national journals increasingly publish at least summaries in English. Nevertheless, further reviews might benefit from searching literature in other languages to provide a more comprehensive overview of the topic, especially for specific populations and races. Second, given that this is a narrative review, we did not examine the methodological quality of the cited studies, and we did not formally evaluate publication bias, as these aspects are mandatory in systematic reviews. Third, the discussion extrapolates findings from a limited and heterogeneous dataset. Nevertheless, we believe that this narrative review could form a basis for further systematic reviews with potential meta-analyses on the relationship between PAD and bone fragility. Namely, the aim of narrative reviews is to identify evidence from heterogeneous datasets to form the basis for defining hypotheses and conducting further systematic reviews with meta-analyses on specific subtopics identified.

## 8. Conclusions and Practical Considerations

Numerous studies have contributed to our understanding of bone fragility determinants in patients with PAD, but countless ambiguities remain to be solved. More detailed research on small-length bone properties (especially on micro- and nano-scale) using a combination of experimental and simulation studies, is required to complete the bone fragility puzzle in patients with PAD. In interaction with clinical data, a multidisciplinary approach to evaluating structural bone hierarchical properties could set a base for developing a patient-specific diagnostic algorithm that reliably predicts fracture risk in these patients. Thus, it is meaningful to evaluate vascular status in individuals with osteoporosis and examine bone health in individuals with PAD to identify individuals who require treatment for both diseases.

From a clinical standpoint, HR-pQCT studies, which are routinely used in some centers in the world, could contribute to improved evaluation of bone health when DXA-based BMD and T score do not provide conclusive evidence. This is particularly true for patients with various comorbidities, in whom T score and BMD may not provide reliable surrogates of fracture risk assessment. However, given the limited availability of HR-pQCT, further methodologies (including DXA add-ons, such as Hip Structure Analysis, Vertebral Fracture Assessment, and Trabecular Bone Score) need to be tested in clinical settings to ensure accurate and timely prediction of individualized fracture risk in patients with PAD.

## Figures and Tables

**Table 1:** Literature search strategy used in our study.

Bone-Related Search Terms	Vascular Search Terms
“bone”	“peripheral artery disease”
”osteopenia”	“PAD”
“osteoporosis”	“peripheral vascular disease”
“bone fracture”	“PVD”
“bone fragility”	“artery calcification”
“bone mineral density”	“aortic calcification”
”bone micro-architecture”	“AAC”
”bone quality”	

The OR search command was used for each type of search term (given in a separate color-coded column), while the AND search command was used between two types of search terms. Bone-related terms are shown in yellow, while vascular search terms are shown in blue. Abbreviations: PAD—peripheral artery disease; PVD—peripheral vascular disease; AAC—abdominal aortic calcification.

**Table 2:** Contemporary studies on bone micro-architectural alterations in individuals with PAD.

Study (Reference)	PAD Assessment Method	Number of Patients	Skeletal Site	Imaging Method	Main Results on Microstructural Bone Properties
Chow J et al. [55]	Agatston score (degree of arterial calcification)	*n* = 693 men, *n* = 321 women, *n* = 372 with PAD, *n* = 550	LS, PF, DR	QCT HR-pQCT	Lower BV/TV and Tb.N, as well as higher Tb.Sp, were associated with AAC in older men, even after multivariable adjustment. Correlations between bone microstructure and AAC in postmenopausal women did not remain significant after multivariable adjustment.
London G et al. [56]	Aortic PWV	*n* = 66 men, *n* = 33 women, *n* = 33 with PAD, *n* = 48	Iliac crest	Optic microscopy	Aortic stiffness and calcification were associated with low bone activity and higher calcium load, suggesting bone–arterial cross-talk in hemodialysis patients with end-stage renal disease.
London G et al. [57]	ABI	*n* = 65 with PAD, *n* = 35	Iliac crest	Optic microscopy	In nondiabetic patients with end-stage renal disease, PAD was associated with osteoblast resistance to PTH and low bone turnover.
Kuipers A et al. [58]	Agatston score (degree of arterial calcification)	*n* = 278 men, *n* = 278 with PAD, *n* = 190	DR, DT	HR-pQCT	AAC was associated with cortical vBMD of the tibia and radius, suggesting a link between cortical remodeling and vascular calcification.
Gaudio A et al. [50]	ABI	*n* = 69 men, *n* = 21 women, *n* = 38 with PAD, *n* = 34	DR	HR-pQCT	Patients with subclinical PAD had significantly reduced cortical density and cortical area, while trabecular micro-architecture in distal radius was not significantly different in comparison to the control group.
Chan J et al. [59]	Agatston score (degree of arterial calcification)	*n* = 1317 women, *n* = 689 men, *n* = 628	LS	QCT	Women and men with low spinal vBMD have greater severity of vascular calcification, particularly at the abdominal aorta.
Paccou J et al. [51]	Lower leg arterial calcifications score	*n* = 341 men, *n* = 179 women, *n* = 162 with PAD, *n* = 111	DR, DT	HR-pQCT	Women with lower leg arterial calcification had lower cortical area and Tb.N, as well as higher Tb.Sp at the distal tibia and lower Tb.Th at the distal radius compared with controls. Men with lower leg arterial calcification had lower Tb.N at the distal tibia.
Atlan et al. [60]	Digito-brachial pressure index	*n* = 33 women, *n* = 33 with PAD, *n* = 12	DR	pQCT	Altered radial micro-architecture in women with systemic sclerosis was associated with the presence of macrovascular disease.

Abbreviations: PAD—peripheral artery disease; AAC– abdominal aortic calcification; DR—distal radius; PF—proximal femur; LS—lumbar spine; HR-pQCT—high-resolution peripheral quantitative CT; QCT—quantitative CT; BV/TV—bone volume/tissue volume; Tb.N—trabecular number; Tb.Sp—trabecular separation; Tb.Th—trabecular thickness; PWV—pulse wave velocity; ABI—ankle-brachial index; PTH—parathyroid hormone; vBMD—volumetric bone mineral density; DT—distal tibia.

## Data Availability

No new data were generated in this narrative literature review. Obtained literature search results supporting claims in this narrative review are available from the corresponding author upon justified request.
